# Supplementary material for: Multifunctional Cotton Fabrics Obtained by Modification with Silanes Containing Esters of Phosphoric Acid as Substituents
Source: Materials (Basel). 2021 Mar 21;14(6):1542. doi: 10.3390/ma14061542 (PMC8004133; doi:10.3390/ma14061542)
Supplement: Supplementary file 1 [file materials-14-01542-s001.pdf]

materials

Supplementary Information

Multifunctional Cotton Fabrics Obtained by Modification with Silanes Containing Esters of Phosphoric Acid as Substituents

Marcin Przybylak [1,*], Michał Dutkiewicz [1,2], Karol Szubert [3], Hieronim Maciejewski [1,3] and Szymon Rojewski [4]

[1] Poznan Science and Technology Park, Adam Mickiewicz University Foundation, Rubież 46, 61-612 Poznań, Poland; midu@amu.edu.pl (M.D.); maciejm@amu.edu.pl (H.M.)
[2] Centre for Advanced Technologies, Adam Mickiewicz University, Uniwersytetu Poznańskiego 10, 61-614 Poznań, Poland
[3] Faculty of Chemistry, Adam Mickiewicz University, Uniwersytetu Poznańskiego 8, 61-614 Poznań, Poland; karolszu@amu.edu.pl
[4] Institute of Natural Fibres & Medicinal Plants, Wojska Polskiego 71b, 60-630 Poznań, Poland; szymon.rojewski@iwnirz.pl
* Correspondence: marcin.przybylak@ppnt.poznan.pl

Citation: Przybylak, M.; Dutkiewicz, M.; Szubert, K.; Maciejewski, H.; Rojewski, S. Multifunctional Cotton Fabrics Obtained by Modification with Silanes Containing Esters of Phosphoric Acid as Substituents. *Materials* **2021**, *14*, 1542. https://doi.org/10.3390/ma14061542

Received: 11 February 2021
Accepted: 16 March 2021
Published: 21 March 2021

Publisher's Note: MDPI stays neutral with regard to jurisdictional claims in published maps and institutional affiliations.

Copyright: © 2021 by the authors. Submitted for possible open access publication under the terms and conditions of the Creative Commons Attribution (CC BY) license (http://creativecommons.org/licenses/by/4.0/).

1. Results of TG Analysis

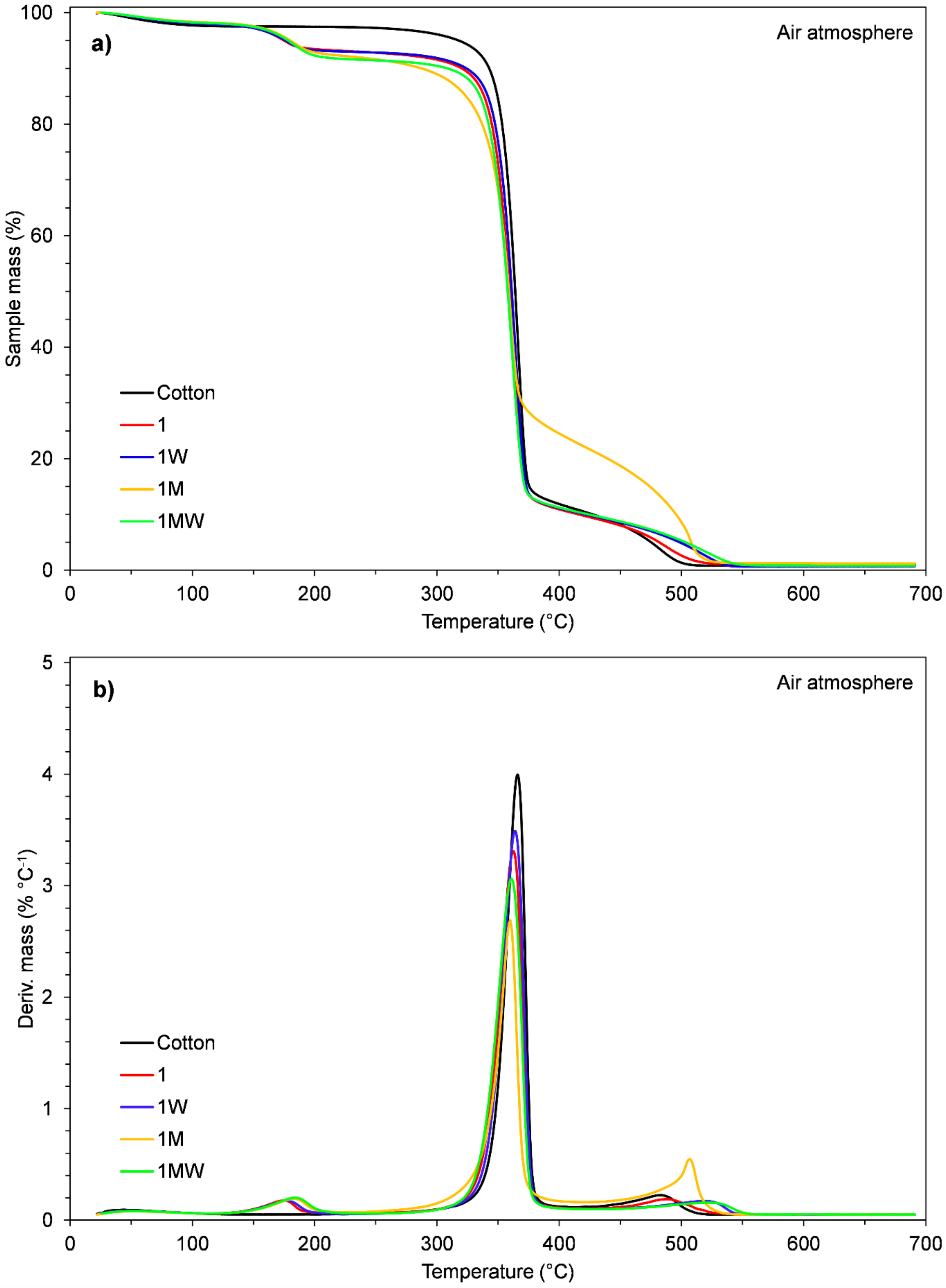

Figure S1. Superimposed TG (**a**) and DTG (**b**) curves of cotton fabric samples modified with prepolymer 1 in single and two-step process before and after five washing cycles.

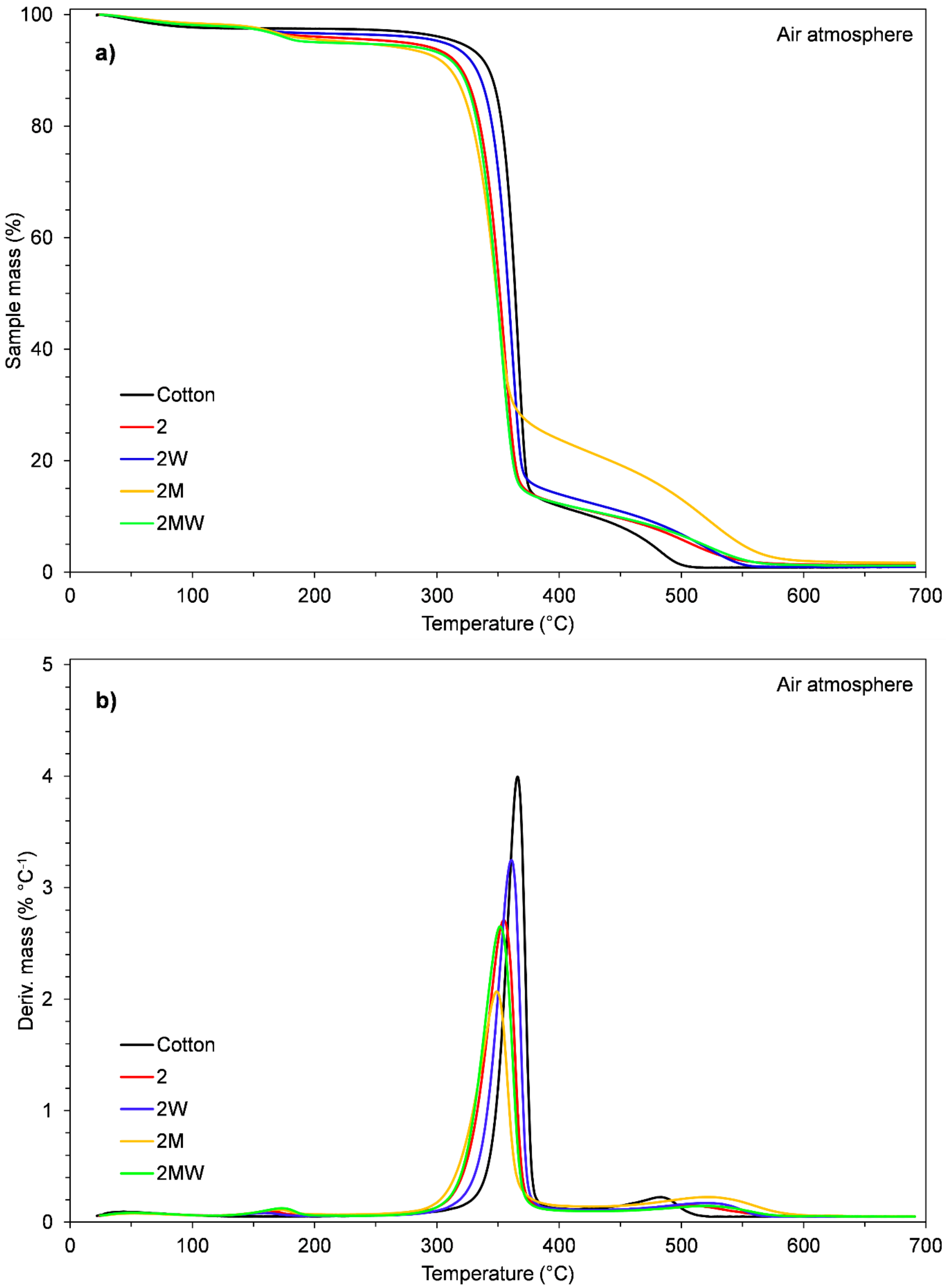

Figure S2. Superimposed TG (**a**) and DTG (**b**) curves of cotton fabric samples modified with prepolymer 2 in single and two-step process before and after five washing cycles.

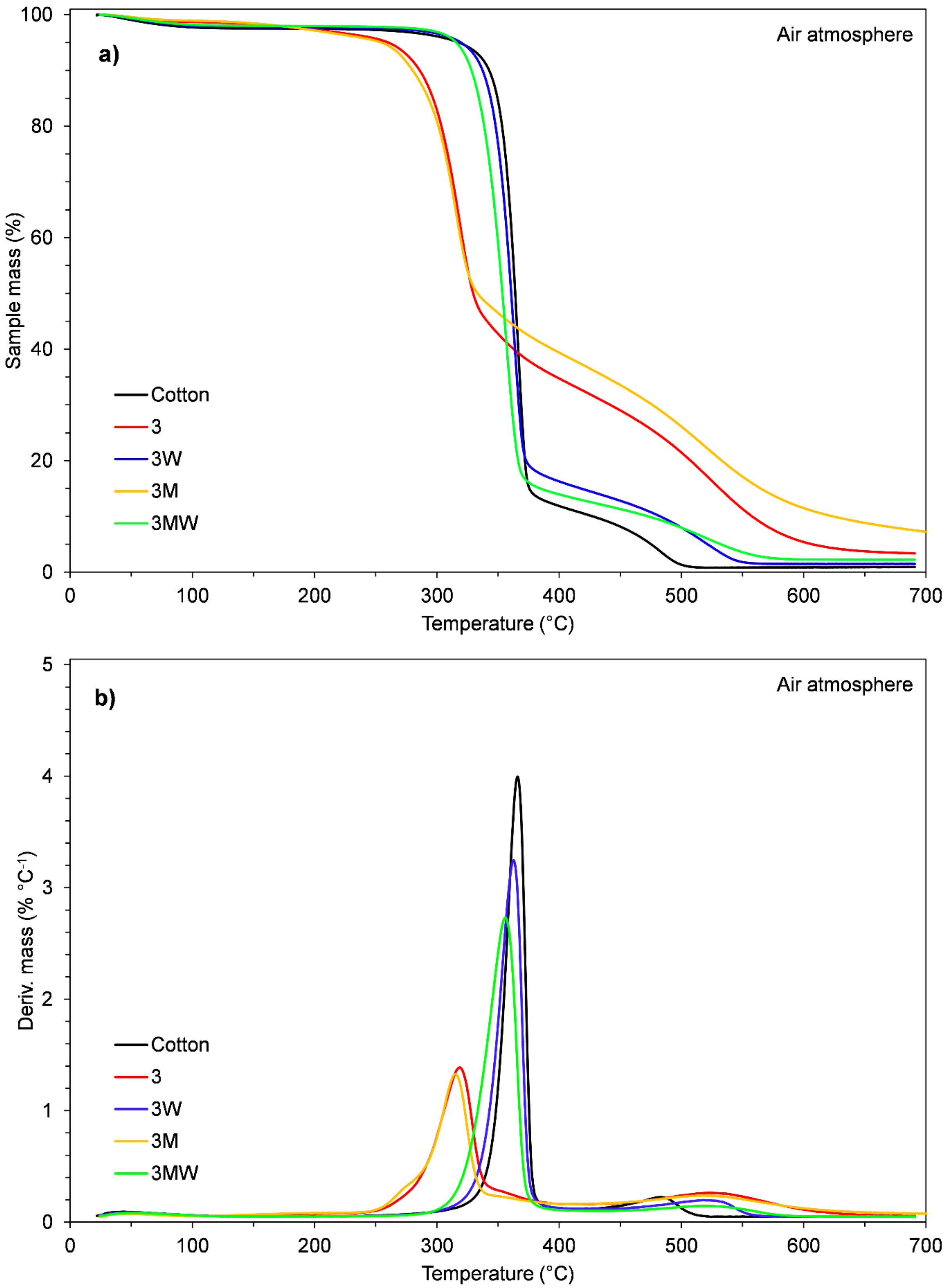

Figure S3. Superimposed TG (**a**) and DTG (**b**) curves of cotton fabric samples modified with prepolymer 3 in single and two-step process before and after five washing cycles.
